# Enhancing Localization Efficiency and Accuracy in Wireless Sensor Networks

**DOI:** 10.3390/s23052796

**Published:** 2023-03-03

**Authors:** Muhammad Fawad, Muhammad Zahid Khan, Khalil Ullah, Hisham Alasmary, Danish Shehzad, Bilal Khan

**Affiliations:** 1 Department of Computer Science & Information Technology, University of Malakand, Chakdara 18800, Pakistan; 2 Department of Software Engineering, University of Malakand, Chakdara 18800, Pakistan; 3 Department of Computer Science, College of Computer Science, King Khalid University, Abha 62529, Saudi Arabia; 4 Department of Computer Science, Superior University, Lahore 54000, Pakistan; 5 Department of Computer Science, FAST-NUCES, Chiniot-Faisalabad Campus, Chiniot 35400, Pakistan

**Keywords:** WSN, localization, energy efficiency, DV-Hop, accuracy

## Abstract

Accuracy is the vital indicator in location estimation used in many scenarios, such as warehousing, tracking, monitoring, security surveillance, etc., in a wireless sensor network (WSN). The conventional range-free DV-Hop algorithm uses hop distance to estimate sensor node positions but has limitations in terms of accuracy. To address the issues of low accuracy and high energy consumption of DV-Hop-based localization in static WSNs, this paper proposes an enhanced DV-Hop algorithm for efficient and accurate localization with reduced energy consumption. The proposed method consists of three steps: first, the single-hop distance is corrected using the RSSI value for a specific radius; second, the average hop distance between unknown nodes and anchors is modified based on the difference between actual and estimated distances; and finally, the least-squares approach is used to estimate the location of each unknown node. The proposed algorithm, named Hop-correction and energy-efficient DV-Hop (HCEDV-Hop), is executed and evaluated in MATLAB to compare its performance with benchmark schemes. The results show that HCEDV-Hop improves localization accuracy by an average of 81.36%, 77.99%, 39.72%, and 9.96% compared to basic DV-Hop, WCL, improved DV-maxHop, and improved DV-Hop, respectively. In terms of message communication, the proposed algorithm reduces energy usage by 28% compared to DV-Hop and 17% compared to WCL.

## 1. Introduction

Wireless sensor networks (WSNs) have garnered significant interest from academia and industry due to their potential use in various applications such as disaster monitoring, target tracking, routing, battlefield surveillance, and many others [1]. Some applications have recently emerged that require the random deployment of many nodes over large hostile environments to be monitored. Based on WSN deployment, we have isotropic and anisotropic categories. In an isotropic network, sensor nodes are uniformly distributed throughout the network with a uniform node density. In contrast, sensor nodes in an anisotropic network are unevenly distributed. If these sensors’ positions are unknown, the data collected is useless until and unless the most feasible exact location can be estimated [2]. Even though several analyses of the relevant literature have been conducted, localization remains a complex and unsolved problem. Developing cost-effective, scalable, efficient, and reliable localization techniques for WSNs has been an active research area. Localization in sensor networks is a significant issue that requires robust solutions.

### 1.1. High-Level Description

**Figure 1:**
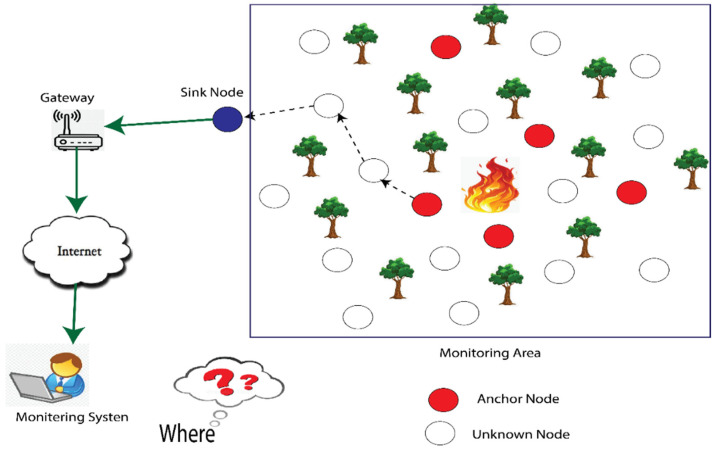
WSN localization.

Localization has received significant attention in recent years. In WSN, some nodes are aware of their location and are referred to as anchor nodes. On the other hand, nodes that do not know their coordinates are called unknown or unlocalized. The coordinates of anchor nodes are calculated using the global positioning system (GPS) [3], whereas unknown nodes estimate their coordinates using anchor nodes, as shown in Figure 1. Many localization algorithms have been developed, but they often face a trade-off between localization accuracy and energy efficiency. High energy costs can impact localization accuracy; conversely, efforts to minimize energy can impact location accuracy. Therefore, it is important to consider accuracy and energy efficiency to achieve a cost-effective and highly accurate localization solution.

### 1.2. Motivation

Numerous localization algorithms have been reviewed and analyzed for accuracy and energy efficiency. These algorithms can be categorized as either range-based or range-free. Some examples of range-based algorithms are AOA, TDOA, TOA, and RSSI schemes [4,5,6]. Alternatively, the location of an unlocalized node can be computed based on Euclidean distance from anchor nodes using algorithms such as DV-Hop [7], a range-free algorithm. Range-free solutions are often preferred in cases where the hardware constraints or energy limitations of sensor nodes require a high degree of localization accuracy. They are also a cost-effective option in such situations.

This paper aims to address the challenges of DV-Hop-based localization in WSNs regarding accuracy, energy efficiency, and position estimation. Ensuring a long network lifespan and high localization accuracy is critical for WSN localization, and determining an accurate and cost-effective localization technique for sensor networks remains challenging. Therefore, there is a need for a mechanism to accomplish energy-efficient and precise localization in WSNs, which motivated this study.

### 1.3. Contribution

This paper proposes an energy-efficient and accurate localization scheme for WSNs. We reduce the number of packets transmitted during the localization phase to improve energy efficiency. We add a step to increase localization accuracy that calculates the accumulated error of the average hop distance (AHD).

The work presented in this paper makes the following research contributions:

- The proposed scheme restricts the broadcasting range or the packets transmitted between nodes by defining a specific threshold that minimizes communication overhead and saves precious network lifetime.
- Introduces a refining step based on the calculation of the AHD error.
- The computational cost of localization time is minimized by introducing a threshold in the proposed algorithm.
- The performance of the proposed algorithm is compared to the state-of-the-art using a MATLAB-based simulator, demonstrating the improvements in energy efficiency and localization accuracy for WSNs.

The remaining sections of the paper are structured as follows: Section 2 examines the current literature on improvements to DV-Hop. The proposed novel algorithm is presented in Section 3. In Section 4, we provide an example of the proposed algorithm. The performance analysis is discussed in Section 5. Finally, Section 6 ends with an overview of the research and suggestions for further study.

## 2. Related Work

Over time, many algorithms to attain energy-efficiency and location accuracy in WSNs have been recommended in the relevant literature. These solutions are divided into range-based and range-free localization approaches [8,9]. For location estimation, the previous approaches employed range or angle measures such as angle of arrival (AOA) [4], time difference of arrival (TDOA) [5], and received signal strength indicator (RSSI) [6]. In contrast, the latter methods used a different localization methodology. In terms of power consumption, hardware cost, and complexity, it has been found that range-free approaches are better than range-based techniques [10]. This part will review some localization techniques based on DV-Hop and its variants.

DV-Hop is a traditional localization method that has gained popularity among academics due to its ease of use, stability, affordability, and minimal hardware requirements. Unknown nodes acquire anchor node information in this algorithm within a defined number of hops and calculate distances between themselves and anchors using DV-Hop [7], as the DV-Hop technique uses the connection information to calculate the shortest paths. Unknown nodes employ the AHD to determine approximated distances to anchors. The three phases of the DV-Hop algorithm are described in the following lines.

Phase 1: Determining Minimum Hop Count by Flooding

**Figure 2:**
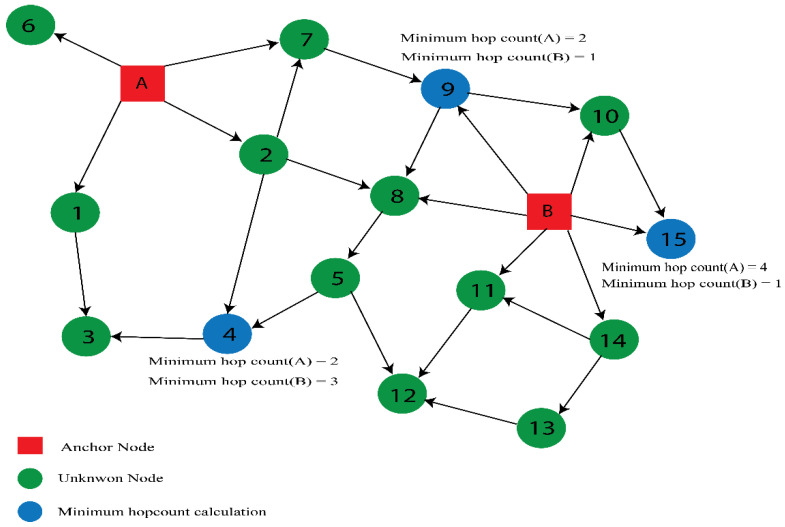
Determining minimum hop count.

All anchors broadcast control packets containing their coordinates, i.e., (Xi, Yi). These coordinates are sent to their neighbors in this stage, and hop values are set to zero. The control packet has the following format: (Xi, Yi, hop value). A neighbor node receives a packet from a specific anchor with a lower hop count, the anchor node’s coordinates are preserved, and its hop value is updated by “1” before forwarding the packet to additional neighbor nodes; otherwise, the message is rejected, as shown in Figure 2. Consequently, each unlocalized node receives the lowest hop count for each anchor.

Phase 2: Determining Distance by Calculating AHD

**Figure 3:**
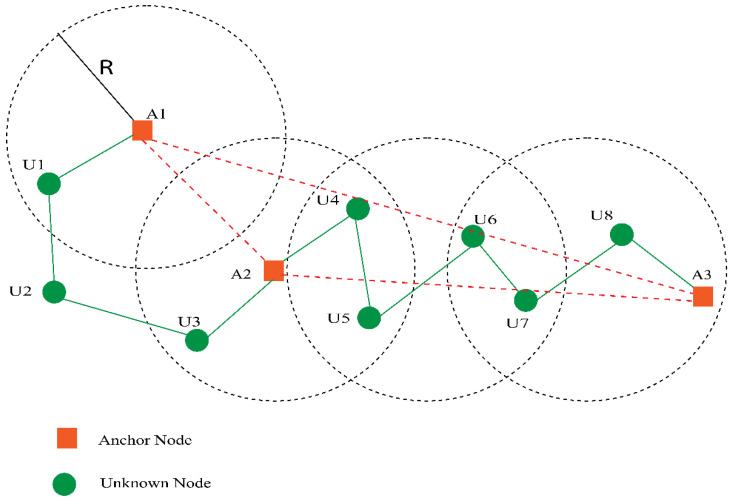
Calculating average hop distance.

In this phase, the anchors receive the minimum hop count computed in the first phase. Then, using the Euclidean formula, the anchor can calculate its distance from different anchors and divide it by the minimum hop count. Anchors determine the AHD of each anchor node in this step, as depicted in Figure 3. The AHD for the anchor node is computed using Equation (1):

(1) $$AHD=\frac{\sum_{i=j}di,j}{\sum_{i=j}hi,j}$$

where $d_{i,j}=\sqrt{{\left(x_{i}-x_{j}\right)}^{2}-{\left(y_{i}-y_{j}\right)}^{2}}$ and the coordinates of anchor nodes *i* and *j* are provided by $\left(x_{i},y_{i}\right)$ and $\left(x_{j},y_{j}\right)$ respectively, whereas the minimum hop value is represented by $h_{i,j}$. After computing HopSize_i_, each anchor node uses controlled flooding to broadcast its HopSize_i_ throughout the system. The approximate distance between the anchor “*i*” and the unlocalized node “*u*” is computed using Equation (2):

(2) du,i=AHD×hu,i

where $h_{i,u}$ is the shortest distance between “*i”* and “*u”*.

Phase 3: Unknown Node’s location Estimate

In this stage, the coordinates of all unlocalized nodes are established. The multi-literation technique [9] approximates the unlocalized node’s position. 

It is presumed that (x, y) are the coordinates of an unlocalized node and (*x_i_*, *y_i_*) are the known coordinates of anchor “*i*”. Let “*d_m_*” be the distance between the unlocalized and the anchor, where m represents the total anchors.

Equation (3) has the following form:

(3) $$\left\{ \begin{array}{l} {\left(x-x_{1}\right)}^{2}+{\left(y-y_{1}\right)}^{2}=d_{1}^{2}\\ {\left(x-x_{2}\right)}^{2}+{\left(y-y_{2}\right)}^{2}=d_{2}^{2}\\ \;\;\;\;\;\;\;\;\;\;\;\;\;\;\;\;\;:\\ {\left(x-x_{m}\right)}^{2}+{\left(y-y_{m}\right)}^{2}=d_{m}^{2} \end{array}\right.$$

The matrix representation of Equation (4) from Equation (3) is as follows:

(4) $${AX}_{un}=B$$

(5) $$A=2\times \left[\begin{array}{ll} x_{m}-x_{1} & y_{m}-y_{1}\\ x_{m}-x_{2} & y_{m}-y_{2}\\ \;\;\;\;\;\;\vdots & \;\;\;\;\vdots \;\;\\ x_{m-1}-x_{m} & y_{m-1}-y_{m} \end{array}\right]$$

(6) $$B=\left[\begin{array}{l} d_{1}^{2}-d_{m}^{2}+x_{m}^{2}-x_{1}^{2}+y_{m}^{2}-y_{1}^{2}\\ d_{2}^{2}-d_{m}^{2}+x_{m}^{2}-x_{2}^{2}+y_{m}^{2}-y_{2}^{2}\\ \;\;\;\;\;\vdots \;\;\;\;\;\;\;\;\;\;\;\;\;\;\;\;\;\;\;\;\vdots \\ d_{m-1}^{2}-d_{m}^{2}+x_{m}^{2}-x_{m-1}^{2}+y_{m}^{2}-y_{m-1}^{2} \end{array}\right]$$

(7) $$X=[\begin{array}{l} x\\ y \end{array}]$$

The unknown node *X* possessing coordinates (x, y) can obtain its calculated coordinates using least-squares methods as follows:

(8) $$X={\left(A^{T}A\right)}^{-1}A^{T}B$$

This scheme will consume more energy due to flooding and position errors due to AHD and will be employed only for isotropic networks. This algorithm still needs improvement with consideration of essential factors such as increasing energy efficiency, improving location accuracy, and reducing communication overhead. The distribution of sensor nodes affects the DV-Hop algorithm’s accuracy; specifically, if the distances between nodes are approximately equal, the average hop size anticipated will be correct, leading to a low localization error. However, the algorithm’s accuracy suffers if the distribution of nodes is unequal [11]. To overcome the drawbacks of the DV-Hop algorithm, a novel method based on the DV-Hop localization technique was proposed by Fang et al. [12]. The RSSI technology is introduced when classifying the current methods based on whether the placement node is one hop away from anchor nodes. Utilizing signal attenuation during signal transit, RSSI calculates the distance. If the sending node’s transmitting signal strength and the receiving node’s received signal strength are known, the signal loss during transmission can be calculated and the formula may then be used to convert the transmission loss to distance. This capability is mostly used in this article so that RSSI technology may find the single hop between two nodes. The other purpose of this technology and the existence of constraints, therefore, had no impact and may be disregarded. Low cost and low power requirements are met via the improved DV-Hop Algorithm. However, external influences can readily impact a signal and affect the ability to ensure that anchor nodes are distributed uniformly and in a specific proportion.

Liu, J. et al. propose various average hop distance (VAH-DV-hop) methods [13] which can reduce energy consumption and eliminate extra hardware. The fundamental ideas of this method are to utilize the angle approach to solve the problems faced by routing and to use varied AHD to improve distance estimation accuracy. According to simulation results, VAH-DV-Hop can boost location precision, especially in networks with uneven coverage. In the DV-Hop method, hop distance is used for straight-line hop distance. However, in a real network, the route between the anchor node and the unknown nodes is not straight. The authors of [14] found that altering the distance between the anchor and unlocalized nodes improved the DV-Hop approach’s accuracy and reduced the localization errors it introduced.

IDVLA, a reliable DV-Hop variant suggested by Chen et al. [15], assesses the average hop size rather than the initial hop size. The least-squares approach was replaced by the weighted least-squares method. Thus, each anchor helps to determine the location of the node. However, if we investigate only a few anchors close to a unlocalized node, there is a greater possibility that the node can be located with more precision. Zhang et al. provide a unique weighted centroid localization (WCL) based on DV-Hop that can only find unknown nodes that are strongly related to the anchor nodes, as illustrated in Equation (9) in [16]:

(9) $$x_{j}=\frac{\sum_{i=1}^{n}w_{i}x_{i}}{n}\;\;\;\;,\;\;\;\;y_{j}=\frac{\sum_{i=1}^{n}w_{i}y_{i}}{n},\;\;\mathrm{where}\;wi=\frac{1}{hopi,j}$$

where (*x_i_*, *y_i_*) are the known coordinates, (*x_j_*, *y_j_*) are the unknown coordinates, and n is the total nodes. WCL consumes more energy, like DV-Hop, and the weight calculation increases computational complexity, which, again, consumes more energy. Furthermore, G. Song et al. introduced the Refined DV-Hop localization technique in [17], which uses a hyperbolic function rather than multiliteration to estimate the average of the average hop size of all nodes. X.Fang et al., in [18], established a technique based on the compensation coefficient that may decrease error by correcting the distances between anchors and unlocalized nodes. 

S. Tomic [19] exploited three advanced DV-Hop variants (iDV-Hop1, iDV-Hop2, and Quad DV-Hop), in which the first two algorithms used the geometry method, which improved accuracy slightly. However, the other algorithm uses a quadratic programming solution instead of the least-squares method to solve non-linear equations. It significantly improves accuracy. As mentioned above, the first two phases in all algorithms are identical and require significant energy due to broadcasting. Energy use can be greatly reduced if this message transmission can be managed somehow. Furthermore, the authors in [20] provide a method for comparing hop sizes to determine the best maximum hop count. The AHD from the source node is modified using this method using a single-hop average error function and a sub-error estimate function. Although employing all anchors reduces inaccuracy, the method requires significant online and offline computing. A non-linear weighted hyperbolic (WH) approach is implemented on each node to acquire its location in [21] by J. Mass-Sanchez et al., which improved accuracy significantly while increasing complexity and processing time. According to the research in [22], a threshold for distance or hop count should be used to improve computation and prevent energy loss from dying nodes.

Xin Qiao [23] proposed a WSN localization technique, based on DV-Hop, which adjusts both the AHD and position computation. The technique utilizes the optimized anchor node information and has better placement precision compared to a single anchor node. The main algorithmic improvements in this technique are the initial value estimate and final estimation of node coordinates. The initial estimate uses the min-max approach when there are fewer anchor nodes, and the ML algorithm is used when there are more anchor nodes. The final estimation’s initial value is repeatedly optimized using the quasi-Newton method. The experiments showed that it provides an efficient and effective ranging-free locating solution for WSN. However, the main benefit of the min-max algorithm is its ability to produce good positioning results with minimal calculation; however, if there are many anchor nodes, its accuracy could suffer.

A technique for enhancing the precision of target localization and tracking in indoor industrial environments was suggested by Khan, M.A., et al. [24]. The method involves selecting dependable nodes by considering the distance between nodes within a cluster and the destination to reduce placement errors. The technique was found to be more accurate in tracking targets than traditional trilateration. However, it is inappropriate for outdoor use or large-scale applications.

D. Xue [25] suggested an improved DV-Hop algorithm based on hop thinning and distance adjustments to mitigate the significant error in DV-Hop. Using RSSI ranging technology, the weighted average of the estimated distance and hop distance errors are utilized to change the AHD and the minimum hop distance. The literature review demonstrated that most efforts have focused on enhancing localization accuracy, whereas energy reduction, a crucial aspect of WSN localization, has not been addressed. Although all the work mentioned above enhances localization precision, relatively few efforts have focused on minimizing energy consumption. Moreover, in [26], the inverse distance weighting (IDW) correction approach produces a precise AHD. Different weights are assigned to anchors based on their distances.

A priority-based algorithm [27] is another strategy that gives a few anchors precedence depending on their AHD. Using high-priority anchors, the weighted centroid approach is then used to locate unknown nodes. Results reveal that the methodology works better in anisotropic fields than the current weighted centroid methods. A second study direction examined the locations of uneven fields. A chaotic environment, network gaps, uneven fields, and irregular node radio propagation patterns are a few anomalies that make node localization challenging [28]. 

An improved version of the DV-Hop algorithm for industrial WSNs, based on the multi-communication radius localization technique and utilizing the cosine theorem to optimize distance estimation for unidentified nodes and correct hop count estimations, was proposed in [29]. The algorithm employs multiple communication radii for broadcasting positions and seeks to minimize the number of hops between unknown and beacon nodes. It then utilizes maximum likelihood estimation to identify the coordinates of the position of the unknown node after modifying hop distance estimations with the cosine theorem. The performance of this improved algorithm is compared to the traditional DV-Hop and DDV-Hop algorithms under varying densities of anchor nodes and communication radii. Results from experiments have shown that the improved DV-Hop algorithm leads to increased location accuracy and a reduction in average location error for unlocalized nodes when compared to the traditional algorithms.

DV-maxHop localization, based on anisotropic and isotropic networks, was proposed by Shehzad et al. [30]. For better network location, the authors included a control parameter named MaxHop. If the hop count exceeds MaxHop, the information from the anchor nodes is ignored by the destination node. This prevents the use of data from different anchor nodes, which leads to distance estimate inaccuracies. The MaxHop technique improves convergence speed, localization precision, and energy usage in both isotropic and anisotropic networks. If anchor nodes are distributed unevenly, the technique either fails to locate all unknown nodes or its precision is significantly reduced. Improved DV-maxHop [31] was proposed in the context of examining the DV-maxHop constraint. In this scheme, we adjust the average hop count of each link between the anchor and unlocalized nodes to rectify the distances using a correction approach. This change, based on the distribution of sensors in the network, enables the sensors to more accurately position themselves. Based on the simulation findings, it is clear that improved DV-maxHop significantly improves location error without adding any additional hardware or increasing communication costs.

Messous, S., et al. proposed a scheme [32] for estimating the distance between unidentified nodes and anchors using the polynomial approximation and the RSSI. In addition, this approach employs a recursive calculation of the localization to increase location estimate precision. Experimental findings demonstrate that this method reduces localization error and enhances localization precision.

In addition to DV-Hop and its other latest algorithms, an energy-efficient strategy is presented by S. Kumar et al. in [33], which suggests that anchors’ hop sizes are calculated and modified at the target node, generating an energy-efficient algorithm. This technique reduces considerable communication between the nodes. Goyat, R., et al. [34] described a three-phase procedure for energy-efficient localization in WSNs. Discovering the one-hop neighbor nodes via beacon nodes by sending additional tone requests and reply packets via the MAC layer to minimize packet collisions is the initial stage. The second stage is to separate the detected one-hop unlocalized nodes into two groups: those with direct and those with indirect communication. This action is taken to increase energy efficiency. To reduce localization errors, a correction factor is then used, and the localized nodes are turned into assistance nodes. In addition, Kaur et al. [35] suggested an approach to investigate how tight anchors affect the results of DV-Hop algorithms, which consume less energy. However, this often leads to overestimating the distance and decreased localization accuracy. Liu et al. [36] reduced hops between unknown and anchor nodes to save energy. Accuracy is achieved by modifying positioned nodes and weighting one-hop distance. Simulations show the above strategy reduces localization rounds, positioning error, and energy consumption. 

This section presents the enhanced version of the DV-Hop algorithm [35], which consists of the three phases outlined below:

- Step 1. The first step is similar to that of a traditional DV-Hop.
- Step 2. All anchors calculate their AHD using Equation (1) and forward it to all other nodes. The unknown node locates the “*t*” anchors nearby, calculates their distance from those “*t*” anchors, and then multiplies the number of hops by the AHD using Equation (2).
- Step 3. Using the least-squares method and only “*t*” nearby anchors, the coordinates of all nodes can be computed using the given Equations (10)–(13).

(10) $$AX_{un}=B$$

(11) $$A=2\times \left|\begin{array}{ll} x_{1}-x_{i} & y_{1}-y_{i}\\ x_{2}-x_{i} & y_{2}-y_{i}\\ \;\;\;\;: & \;\;\;\;:\\ x_{t-1}-x_{t} & y_{t-1}-y_{t} \end{array}\right|$$

(12) $$B=\left|\begin{array}{l} x_{1}^{2}-x_{t}^{2}+y_{1}^{2}-y_{t}^{2}-d_{1t}^{2}-d_{unt}^{2}\\ x_{2}^{2}-x_{t}^{2}+y_{2}^{2}-y_{t}^{2}-d_{2t}^{2}-d_{unt}^{2}\\ x_{t-1}^{2}-x_{t}^{2}+y_{t-1}^{2}-y_{t}^{2}-d_{t\left(t-1\right)}^{2}-d_{untt}^{2} \end{array}\right|$$

The unknown node *X* with coordinates (x, y) can calculate its estimated coordinates using least-squares methods as follows:

(13) $$X={\left(A^{T}A\right)}^{-1}A^{T}B$$

From the literature review, it is evident that when we improve localization accuracy, energy consumption increases. On the other hand, location accuracy is compromised if we want to minimize energy consumption. While previous research has made some progress in improving localization accuracy in some studies [7,12,16,25,30,31,32] and reducing energy consumption in others [33,35,36], there is still room for further improvement. To the best of our knowledge, no studies have been focused on minimizing the trade-off between energy efficiency and localization accuracy. Thus, there is a dire need for a contemporary solution in WSN localization to obtain higher localization accuracy while focusing on minimized energy consumption. These techniques allow a certain degree of localization error reduction, but there is still room for improvement.

## 3. The Proposed Enhanced DV-Hop Algorithm

In this paper, we propose the Hop-correction and energy-efficient DV-Hop (HCEDV-Hop) algorithm, an enhanced version of DV-Hop, to enhance location accuracy and energy efficiency. The HCEDV-Hop algorithm aims to exclude anchor nodes that could cause significant errors in the AHD calculation by setting a range error factor. This improves the accuracy of AHD calculations and reduces the impact of random topology. Next, the anchor node broadcasts the corrected AHD to the t-hop threshold to prevent unknown nodes from receiving information from all anchor nodes. The algorithm then calculates the distances between the anchor and unlocalized nodes based on the corrected AHD for t hops. It approximates these distances using the least-squares method to enhance location accuracy as shown in Algorithm 1.**Algorithm1** HCEDV-Hop WSN Localization with Correction step This study introduces an enhanced version of the DV-Hop algorithm named Hop-correction and energy-efficient DV-Hop (HCEDV-Hop). We demonstrate that this algorithm can accurately predict the locations of unlocalized nodes with low energy consumption by introducing a threshold and correcting the AHD. **Input:** Total nodes n, Anchor nodes *m*, coordinates (X_i_, Y_i_), communication range *R*, area 500 * 500 m^2^ **Output:** Location estimate *X_n_* of unknown nodes **Initialization: i** = 1,2, 3, …, n Packet = 0 *Selecting a set of anchors for the localization procedure*
 for(i=2 to n)
 for(j=1 to i−1)
 $d_{i,j}=\sqrt{{\left(x_{i}-x_{j}\right)}^{2}+{\left(y_{i}-y_{j}\right)}^{2}}$
 ***Packet = Packet + 1***
 $if\;\left(d_{i,j}<=R\;\right)$
   hop=1
 else
   hop=hop+1
 end
 hij=hji
 end for 
 end for
 for(i=1 to n−m)
 ***Packet = Packet + 1***
 if (hop==1)
   *Using RSSI by Equation (14).*
 else
   finding minimum hop
 end
 **Calculate AHD using Equation (16)**
 **Calculate the AHD error using Equation (17)**
 **Calculate distance by adding error using Equation (18)**
 **Position estimation Xn of the unknown node using Equation (10)–(13) for t-specific threshold**
 end for
 ***Estimate coordinates of unknown node n and energy consumption in terms of a packet exchanged*** **End**

Design Goals

In the DV-Hop localization technique, the distance between unlocalized and anchor nodes is calculated using an unbiased estimate, which can introduce errors in the AHD calculation, such as cumulative errors in the AHD and unknown node estimates. We propose an AHD removal step in the HCEDV-Hop algorithm to minimize these errors. This enhances the accuracy of the AHD and unknown node estimation calculations in the final step of the algorithm. 

Flooding is a resource-intensive and energy-demanding process that generates significant communication overhead. In the first phase of the proposed algorithm, anchor nodes transmit information data packets which are then forwarded by neighbor nodes, increasing the hop value by one and saving the anchor node’s information. The information is then sent on to other nodes rather than back to the source. During the flooding communication stage, an unknown node may receive the same data from multiple anchor nodes through different routes. The unknown node saves the information data that have traveled the minimum number of hops, which minimizes resource and energy consumption.

**Figure 4:**
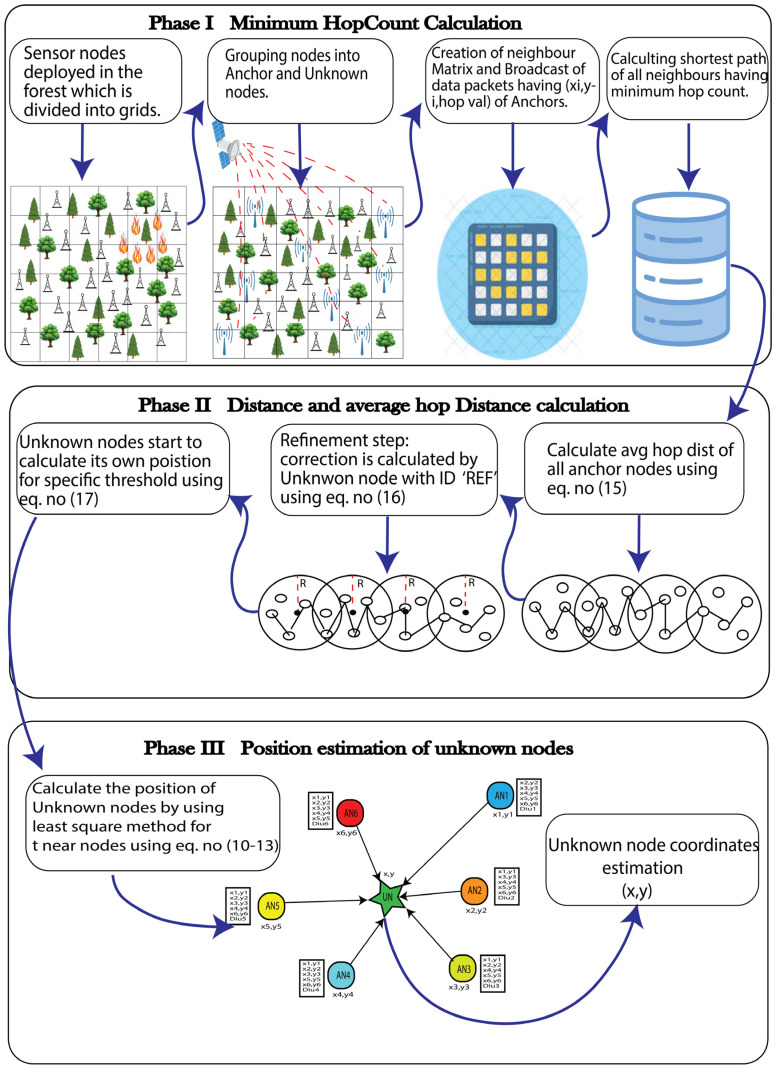
Overall architecture of the proposed algorithm.

The overall architecture of the HCEDV-Hop algorithm is depicted in Figure 4.

The proposed solution, similar to DV-Hop, comprises three phases, but the second step includes a refinement sub-step.

Phase 1. Minimum Hop Calculation

The localization of unidentified nodes is initiated by anchors. To initiate localization, each anchor specifically sends a begin anchor msg (BAG) message to its neighbors. The BAG message consists of the fields (N_ID, coordinates, hop value), where N_ID is the node identification, coordinates are the node’s location, and hop value is the node’s hop count. Each node that receives this message evaluates its location before forwarding it to its neighbors. By employing this method, we guarantee that the closest nodes to the anchor nodes are localized first and sequentially. Every node that receives this message stores the identifier and approximate position of its neighbor. Once a node has at least three anchors and/or neighbors, it may use multiplication to determine its location.

Recall that each node has a table named H_Table that includes the hop value and the coordinates of every other node. Phases 1 and 2 of this study provide the data. The N_ID and the coordinates of the neighboring node are stored in the H_Table, and this table is updated whenever a BAG message is received from a neighbor.

To minimize message forwarding and energy consumption and increase network lifespan, the last two phases of the proposed algorithm utilize controlled flooding or thresholding.

Phase 2. Distance Calculation

If the anchor and unknown nodes have just one hop value, distance estimation is performed using the *RSSI* approach, as suggested by L. Girod et al. in [36] using Equation (14).

(14) $$d=10\frac{A-RSSI}{10n}$$

where *d* is the distance, *A* is the transmitted power at the transmitter node, *RSSI* is the transmitter power at the received signal, and *n* is the attenuation constant. 

The *RSSI* method involves anchor nodes sending beacons to every nearby node in the given dataset, and the neighbors respond with a beacon containing signal strength data. Using the RSSI value doesn’t call for any specialized hardware or add extra expenses because the MAC sub-layer in the majority of current WSNs computes and transfers the *RSSI* value for every received packet to higher layers. The *RSSI* can be calculated using Equation (15):

(15) $$RSSI=A-10n\log_{10}^{\frac{d}{d_{0}}}+X_{\sigma }$$

Theoretically, with path loss assumed to be negligible, the signal intensity is proportional to the distance between anchors and adjacent nodes. From this information, the distance of an anchor node to a neighboring node can be calculated for a single hop.

Each anchor *i* calculates its approximate AHD under a specific threshold using Equation (16) to minimize the amount of energy used:

(16) $$AHD_{i}=\frac{\sum_{i=1\;\;\;i\neq j}^{m}\sqrt{{\left(x_{j}-x_{i}\right)}^{2}+{\left(y_{j}-y_{i}\right)}^{2}}}{\sum_{i=1\;\;\;i\neq j}^{m}hji}\;$$

In WSN, nodes are deployed randomly, resulting in non-linear paths between them that can cause the AHD to be larger than the actual value and introduce errors in estimation. Improving the precision of the AHD increases the accuracy of estimated locations. In the proposed algorithm, we introduce a refinement step in AHD calculation under threshold “t” to minimize errors in estimated positions and improve localization accuracy, rather than using broadcast communication. This is achieved by defining a new formulation for AHD calculation in this phase.

In a WSN where nodes are deployed randomly, the AHD (which represents the distance between nodes) is not a straight line and can deviate significantly from its actual value. In contrast, the Euclidian distance formula is applied to a straight line. This leads to large errors when the AHD error is multiplied by the hop count value.

Each anchor is able to determine its own refine error value as a result of the error correction formula, which has the consequence of effectively reducing the cumulative error when identifying unknown nodes. It is important to note that the AHD of all anchors are correct to a sufficient degree in the scenario being discussed, and that the best possible solution has been found. To reduce this error, the AHD is corrected by subtracting it from the communication radius and multiplying the result by the hop count value before dividing the full expression by the communication radius. This correction results in a small difference between the real and estimated coordinates, thereby improving accuracy under a specific threshold. In our simulation, we varied the threshold value from three to eight. We determined the most appropriate threshold value through iterative execution of the proposed algorithm to achieve the desired accuracy. The error calculation in the second phase of DV-Hop, as shown in Equation (17), can be fine-tuned to improve accuracy.

(17) $$REF=\frac{\left(HC*\left(R-AHD_{i}\right)\right)}{R}$$

The correction term (*REF*) and the anchor’s minimum hop count (*HC*) from an unknown node u, as well as the communication range (*R*), are used to calculate the refined AHD value for each anchor under a specific threshold. The network broadcasts this refined AHD value. The distance (*d_i_*) between “*i*” and “*u*” within a specific threshold is then determined using Equation (18):

(18) $$d_{i}=\left(HC*AHD_{i}\right)+REF$$

Phase 3. Unknown Node Position Estimation

In the final stage, the unknown node improves the accuracy of its estimated coordinates using the least-squares method for a specific threshold “t”, as shown in the Equations (10)–(13). The proposed solution reduces energy consumption and improves localization accuracy by minimizing errors. 

## 4. Example Scenario of the Proposed HCEDV-Hop Algorithm

**Figure 5:**
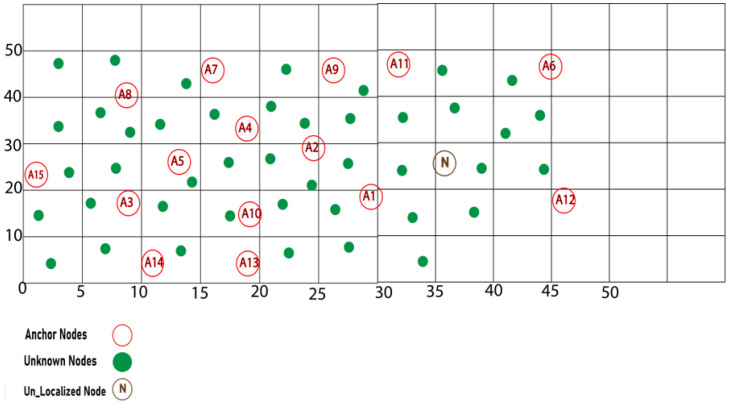
Example scenario of proposed algorithm.

In an WSN with a 50 * 50 m^2^ region, 15 anchors and 35 unknown nodes are dispersed randomly with a communication radius of 10 m, as depicted in Figure 5. Suppose we wish to discover the position of a particular node “N” in the WSN grid with actual coordinates 〈36.24 28.81〉. 

Node “N” follows the steps below to estimate its coordinates: 

Phase 1. The first stage is identical to the DV-Hop process. As indicated in Table 1, the anchor nodes provide information to all other nodes. Table 2 provides the minimum hop value of a node “N” from each anchor node.

Phase 2. Equation (16) is used in this phase to compute the AHD of all anchor nodes under a specific threshold. The AHD for each network anchor is shown in Table 3. Then, we calculate the AHD correction using Equation (17), which is shown in Table 4. After calculating the AHD from each anchor, Node “N” adds the refinement and calculates the distance under a specific threshold, as illustrated in Equation (18). The distance between node “N” and each anchor is depicted in Table 5.

Phase 3. The position is estimated using the least-squares approach for a given threshold with six hops to locate the node “N” location.

The coordinates of unknown nodes can be calculated using Equation (10), where

(19) $$A=2\times \left|\begin{array}{ll} x_{1}-x_{i} & y_{1}-y_{i}\\ x_{2}-x_{i} & y_{2}-y_{i}\\ \;\;\;\;: & \;\;\;\;:\\ x_{5}-x_{6} & y_{5}-y_{6} \end{array}\right|$$

(20) $$B=\left|\begin{array}{l} x_{1}^{2}-x_{6}^{2}+y_{1}^{2}-y_{6}^{2}-d_{6}^{2}-d_{un6}^{2}\\ x_{2}^{2}-x_{6}^{2}+y_{2}^{2}-y_{6}^{2}-d_{12}^{2}-d_{un6}^{2}\\ x_{3}^{2}-x_{6}^{2}+y_{3}^{2}-y_{6}^{2}-d_{18}^{2}-d_{un6}^{2}\\ x_{4}^{2}-x_{6}^{2}+y_{4}^{2}-y_{6}^{2}-d_{24}^{2}-d_{un6}^{2}\\ x_{5}^{2}-x_{6}^{2}+y_{5}^{2}-y_{6}^{2}-d_{30}^{2}-d_{un6}^{2} \end{array}\right|$$

Solving Equation (10) produces node “N” coordinates much closer to the actual values. 

The proposed algorithm can be used for various applications. Possible application scenarios (static anchor and unlocalized static nodes) include the case in which a WSN is deployed in an industry/factory to monitor the temperature and vibration of different machines and equipment. The WSN is composed of anchor nodes and unlocalized nodes. The anchor nodes could also be used for other purposes, such as providing power to the unlocalized nodes, or acting as routers to relay data from the unlocalized nodes to the central server. These anchor nodes have GPS or other external localization capabilities and serve as reference points for the localization of the unlocalized nodes. They act as gateways to the central server and are used to communicate with the unlocalized nodes, supplying the essential information for calculating their positions. The unlocalized nodes could be placed on or near a wide range of machines and equipment, such as pumps, motors, and conveyor belts, depending on the specific needs of the factory, to measure temperature and vibration. These unlocalized nodes do not have GPS or other external localization capabilities, and they communicate with the anchor nodes using wireless signals. The unlocalized nodes are not linked to the central server; therefore, they communicate sensor data to the anchor nodes, which utilize the method to determine the unlocalized nodes’ positions based on hop count information and wireless signal RSSI. It uses a distributed technique in which each network node is able to perform localization computations. In the first phase, each node determines its minimum hop value, stored in the H_Table. In the second phase, if the hop value is one, then its distance is measured by RSSI using Equation (14) otherwise, the AHD is calculated using Equation (16) and then the refinement step is performed using Equation (17). Based on this refined AHD, the algorithm uses the refined AHD to compute the distance between the unlocalized node and the anchor nodes by using Equation (18) in the last phase. This distance is then used to estimate the location of the unlocalized node by multilateration. Once the algorithm has converged and estimated the unlocalized node’s position, the anchor nodes transmit the localization information to the central server. The central server could use this information to provide real-time monitoring and analysis of temperature and vibration in the factory, as well as historical trend analysis. The central server could also incorporate machine learning models to analyze the sensor data, automatically identify patterns in the data, and notify the factory operators of potential issues. The detailed location and sensor data can provide valuable insights for the maintenance and operation of the factory.

Overall, this scenario describes a wireless sensor network that can be used to monitor the conditions of the machines and equipment in an industrial factory. The WSN can be configured to handle the specific needs of the factory and the proposed algorithm can be implemented in a factory environment to accurately locate unlocalized nodes, therefore providing valuable insights for maintenance, operation, and predictive maintenance. It is worth noting that the proposed algorithm assumes that the network is static and that the anchor nodes and unlocalized nodes are placed at fixed locations. As with any localization algorithm, the accuracy of the proposed algorithm depends on the quality of the input data (hop count and RSSI measurements) and the environment; therefore, it may require some form of calibration and fine-tuning to achieve optimal performance in a specific environment.

## 5. Implementation and Simulation Setup

This section assessed the evaluation metrics and simulation setup with related parameters under several conditions. Simulation results are produced and elaborated on in the following section.

### 5.1. Network Model and Setup

**Figure 6:**
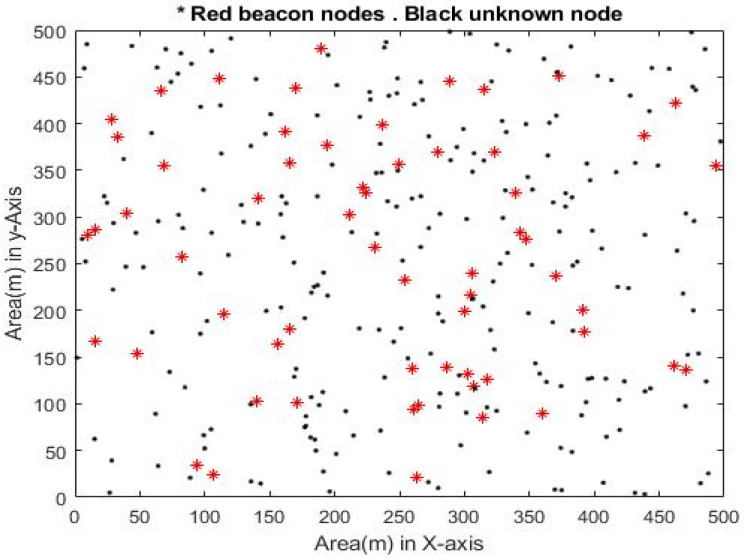
Schematic diagram of randomly generated WSN.

In the simulation, N-*m* unknown nodes and “*m*” anchor nodes are dispersed in a WSN with a 500 * 500 m^2^ area, with the option of deploying 500 sensor nodes, which uses the random function to construct network topology, as shown in Figure 6. The diagram’s 50 red stars (“*”) represent 50 anchor nodes, whereas the 450 black points (“.”) represent unknown nodes. It is assumed that the network is ideal, i.e., all nodes can communicate freely.

Our proposed algorithm is an enhancement of the DV-Hop localization algorithm for WSNs, using hop count information to estimate node distances and determine the location of unlocalized nodes. However, the DV-Hop algorithm can be implemented on top of various wireless standards and protocols that are used for WSNs. It can be implemented on various wireless standards and protocols for WSNs, including IEEE 802.11 and Z-Wave.

### 5.2. Wireless Channel

A realistic wireless connection model is required for a reliable evaluation. The most typical model used to represent the shadowing path loss effect is a log-normal model, as indicated in Equation (21), which results in the following:

(21) $$P_{l}{\left(d\right)}^{B}=P_{l}{\left(d_{e}\right)}^{B}+10_{y}\log\left(\frac{d}{d_{e}}\right)+X_{0}^{B}$$

The power loss after the signal has traveled through distance *d* is represented by *P_l_(d)^B^*, with *P_l_(d_e_)^B^* as the power loss at the reference distance *d_e_*. The path loss exponent is represented by ^γ^, while *X_σ_* is a Gaussian random variable with a mean of 0 and standard deviation σ that accounts for shadowing effects.

### 5.3. Assumption

Localization methods such as DV-HOP and our proposed algorithm assume a spherical radio range; however, when the radio range becomes irregular, the performance of such protocol declines. In the event of radio irregularities, they may not be able to ensure complete coverage, and blind areas may result. As a result, we used an isotropic WSN, based on the spherical radio spectrum, in place of an irregular WSN and we chose the regular model rather than the RIM model [37].

### 5.4. Parameters Setting

The simulation settings employed in the experiment are shown in Table 6. The selected area, i.e., 500 * 500 m^2^, is selected for a fair comparison with the algorithm chosen in the previous research studies [16,35].

### 5.5. Simulation Setup

The performance of the proposed method, in terms of localization accuracy and energy usage, was assessed by an Intel(R) Core(TM) i5-5200U, 2.20GHz CPU PC with 8 GB RAM using MATLAB 2020a [38]. The outcomes were compared with DV-Hop [7], WCL [16], improved DV-maxHop [31], and improved DV-Hop [35]. For each simulation, we performed 50 iterations (with a distinct topology for every iteration) and plotted the average results.

### 5.6. Performance Analysis

Localization accuracy and cost metrics were considered while evaluating and analyzing the HCEDV-Hop algorithm. Comparing the outcomes of the proposed method with DV-Hop, WCL improved DV-maxHop and improved DV-Hop by programming with their descriptions in [16,31,35].

The following metrics evaluate the performance analysis.

#### 5.6.1. Accuracy Metric

Localization accuracy can be defined based on localization error, which is the difference between actual and calculated location. The accuracy is tested by adjusting parameters such as average localization error, anchor ratio, and node density.

The optimal value of “*t*” for the proposed algorithm

**Figure 7:**
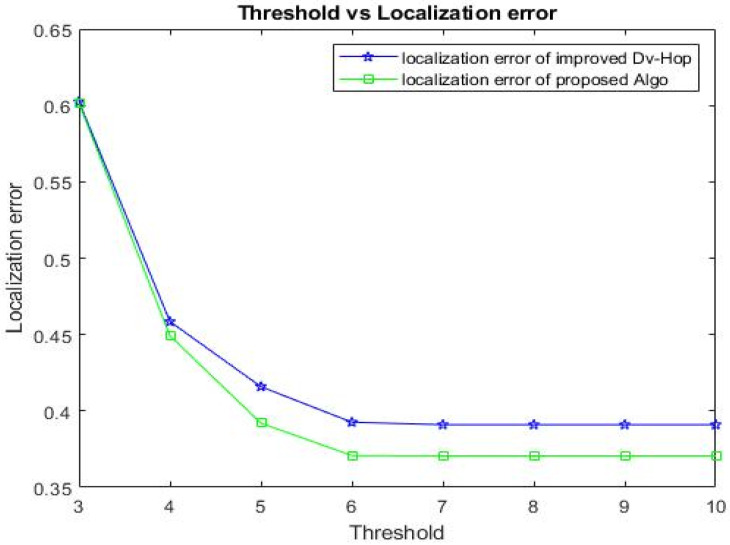
Comparison of threshold with/without using correction step.

In the WSN area, 100 anchors are deployed to ascertain the value of “*t*”. As “*t*” approaches six, Figure 7 shows how the localization error becomes constant.

The accuracy metric is evaluated under the following:

1. Average localization error analysis;
2. Impact of anchor ratio;
3. Effect of varying node density.

##### Average Localization Error Analysis

The average localization error (ALE) [39,40] is the sum of localization errors for unknown nodes. However, accuracy should also consider the number of nodes; hence, the ALE determines the implemented algorithm’s accuracy level. Equation (22) is used to calculate the ALE, which is used as the evaluation criterion:

(22) $$ALE=\frac{\sum_{i=1}^{n}\sqrt{{\left(x_{i}-x\right)}^{2}+{\left(y_{i}-y\right)}^{2}}}{n\times R}$$

The numerator in Equation (22) reflects the Euclidean distance [41] between the predicted (*x*, *y*) and actual locations (*x_i_*, *y_i_*) of the unknown node, as well as the calculated error distance; *n* is the total number of nodes and *R* represents the radius. 

The algorithm proposed in this study enhances localization accuracy by reducing the localization error, as depicted in Table 7, in terms of maximum, minimum, average, and standard deviation. 

**Figure 8:**
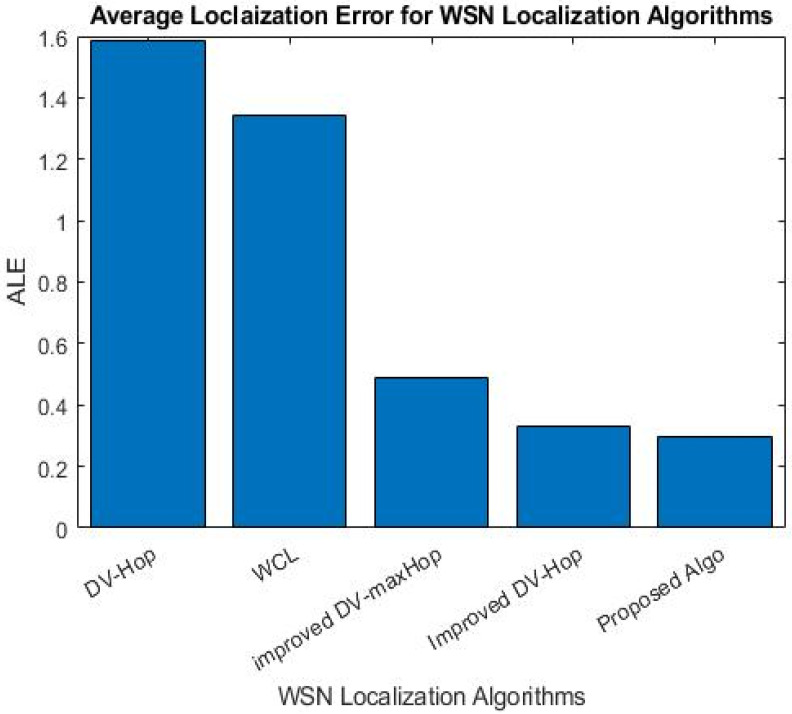
ALE for WSN localization algorithms.

In terms of minimum, maximum, and average, the suggested method outperformed the other three localization algorithms average in terms of average localization error. Figure 8 shows that the proposed algorithm achieved significantly lower ALE compared to DV-Hop, WCL, improved DV-maxHop, and improved DV-Hop, with reductions of 81.36%, 77.99%, 39.72%, and 9.96%, respectively.

##### Impact of Anchor Ratio

The anchor ratio is the proportion of anchor nodes participating in the localization process. While maintaining the total number of nodes and the communication radius at 500 and 100 m, respectively, the localization error decreases as the anchor ratio increases. Table 8 presents the empirical analysis of the average localization error under various anchor node ratios. 

**Figure 9:**
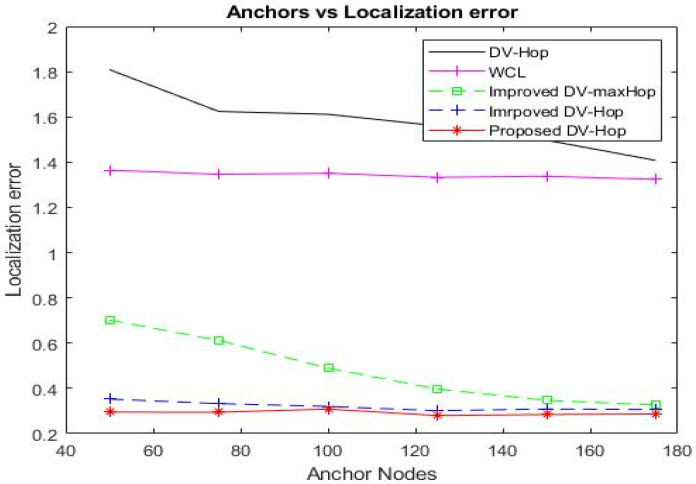
Simulation results for localization error using various anchor ratios.

As the proportion of anchor nodes participating in the localization process increases from 100 to 175, the improved DV-Hop algorithm reduces the localization error from 0.3528 to 0.3066. Our proposed approach decreases the localization error from 0.294917 to 0.289721, as depicted in Figure 9.

When the total number of anchors is 175, the HCEDV-Hop algorithm performs the best, with the lowest error rate, compared to DV-Hop [7], WCL [16], improved DV-maxHop [31], and improved DV-Hop [35], resulting in a reduction in error of 79.41%, 78.11%, 11.26%, and 5.5%, respectively. As the number of network anchors increases, localization accuracy improves. However, using GPS to determine the positions of anchors beforehand can be expensive and energy-consuming. When the anchor node ratio increases, the number of neighbor nodes within the communication radius and hop size increases, tending towards the actual value.

It is evident from Table 8 that the anchor node ratio is between 50 and 100, and that the localization error of our proposed algorithm, HCEDV-Hop, differs significantly from that of the improved DV-Hop algorithm. However, this difference decreases slightly when the anchor node ratio exceeds 100. Our proposed algorithm, HCEDV-Hop, exhibits a very low localization error, performing significantly better than the other algorithms.

##### Impact of Varying Node Density

**Figure 10:**
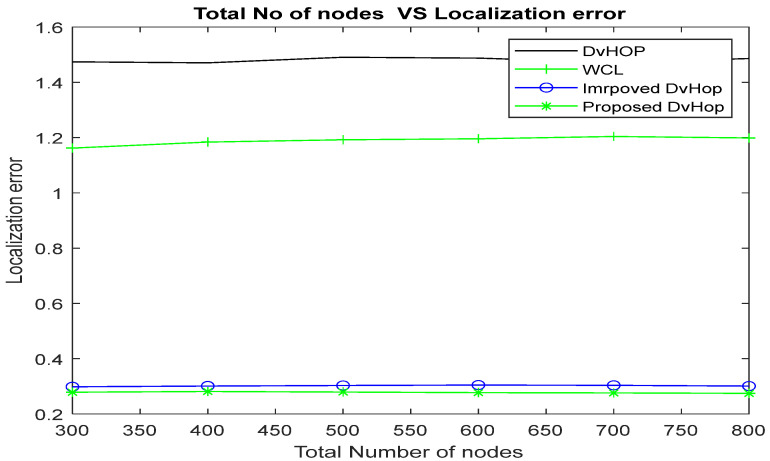
Simulation results of ALE using various total numbers of nodes.

During simulation, the number of nodes steadily rises from 300 to 800, and the number of anchor nodes is set at 50. Figure 10 and Table 9 list the empirical outcomes of the average localization error for various numbers of nodes. 

The proposed algorithm demonstrated a decrease in localization errors compared to DV-Hop, WCL, and improved DV-Hop when the total number of nodes was 300, with reductions of 80.4%, 75.14%, and 1.64%, respectively. As the total number of nodes increased beyond 800, the proposed algorithm continued to outperform traditional DV-Hop [7], WCL [16] and improved DV-Hop [35] by 80.17%, 75.41%, and 2.2%, as depicted in Figure 10.

The localization error reduces as the number of sensor nodes grows. The increase in sensor node density leads to an increase in the number of one-hop neighbors, which improves network connectivity.

The proposed algorithm demonstrates improved localization accuracy and energy efficiency performance compared to other methods. We could minimize communication costs and extend network lifetime while improving localization accuracy by introducing a threshold in the final two phases and adding a correction step. The results clearly show that the proposed algorithm outperforms the others.

##### Impact of Varying Coverage Area

The coverage area of a sensor field refers to the geographical area or region that is covered or monitored by the sensors in the field. In the simulation, we deployed a coverage area from 100 to 900 and analyzed its impact on average localization error. While maintaining the total number of nodes and communication radius at 300 and 100 m, respectively, the localization error increases as the coverage area increases.

Table 10 shows the relationship between coverage area and average localization error. The ALE increases as we move from denser to more sparse coverage, due to high hop value and a decrease in the connectivity between different nodes. The results prove the validity of our proposed algorithm, which obtained a lower ALE than its counterparts. In sparse networks with poor connectivity, it may be difficult to accurately locate certain isolated nodes.

The overall summary of this section is:

- As the ratio of anchor nodes increases, there are more anchor nodes in the deployment region; thus, the AHD of anchor nodes is more precise.
- As the number of deployed nodes increases, the dispersion of nodes tends to become denser and more concentrated in dense networks. As each node is connected to more nodes through a single hop, network connectivity improves. Overall, as the number of deployed nodes increases, the ALE of nodes reduces, generating a more efficient network.
- In denser networks, the ALE tends to decrease due to better connectivity between nodes. However, in sparser networks, the ALE tends to increase due to poorer connectivity between nodes. This occurs as a result of a decrease in connectivity among sensor nodes when the sensing field grows. As a result, some isolated nodes may be unable to be accurately located, leading to an overall increase in the ALE.

#### 5.6.2. Average Energy Consumption

Energy is mostly utilized in three phases by localization solutions: packet transmission, packet reception, and localization calculations. There are various methods to determine the consumed energy. Regardless, we only looked at the cost of communication based on the number of data or control packets exchanged inside the network during the localization process. Communication is widely considered the network’s most power-intensive resource. Calculating the communication cost is necessary to assess the energy consumption involved in localizing the whole network. The proposed algorithm’s energy model is the same as [42,43,44]. We neglect the energy spent during calculating, listening, and sleep stages since it is little compared to the energy consumed during transmission and reception [45]. The majority of the energy is used up by the transmission of data packets from one node to another. In the first phase of our proposed algorithm, the anchor determines the minimum hop count, and AHD packets are the same as in IEEE 802.11. As a result, by limiting packet transmission between nodes, energy consumption may be minimized. Equation (23) may be expressed for DV-Hop as in [7]:

(23) Energy−consum=2×(n−1)×m×E

where *n* represents the total number of nodes, *m* represents the total number of anchor nodes, and *E* represents the average energy utilized to send a packet. Hence, packet transmission occurs in two stages multiplied by two. On the other hand, in our proposed solution, energy is minimized due to specific threshold value generation instead of broadcasting to the whole network, as shown in Equation (24):

(24) Energy−cons=2×(n−1)×(m−t)×Eavg

where *E_avg_* denotes the average energy inside a *t* hop, *m* represents the total number of anchor nodes, and 2 indicates packet transmission in two phases. The total number of sent and received packets (TSRP) is computed to determine the communication cost of all techniques.

Table 11 displays the TSRP for all methods, where “*n*” denotes the total number of network nodes, “*m*” represents the number of anchor nodes, and C_avg_ indicates the average connectivity.

In our simulation, the “300” total number of nodes is dispersed in a WSN with a 500 * 500 m^2^ area. A single iteration of the simulation was performed to compute the result, setting the threshold from 3 to 6 with a communication radius of 100 m.

**Figure 11:**
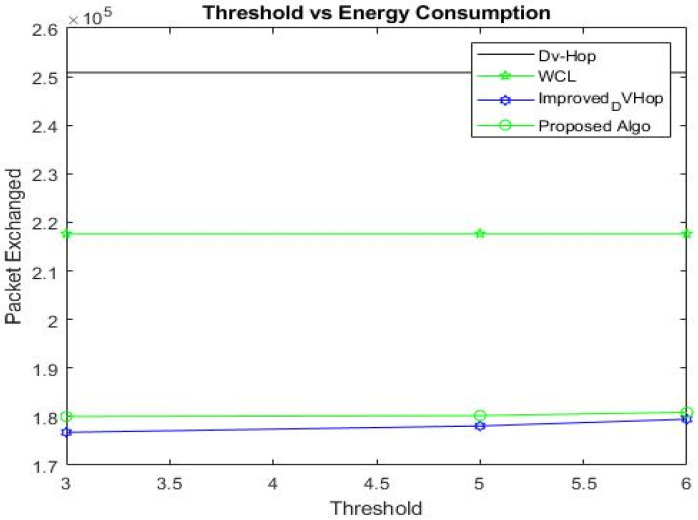
Threshold vs. energy consumption.

The proposed algorithm demonstrates superior energy efficiency compared to DV-Hop, WCL, and improved DV-Hop when t = 3, 5, and 6. Specifically, it reduces energy consumption by 28.24% compared to DV-Hop, reduces it by 17.30% compared to WCL, and slightly increases it—by 1.8%—compared to improved DV-Hop, when t = 3; reduces energy consumption by 28.1% compared to DV-Hop, reduces it by 17.21% compared to WCL, and somewhat increases it—by 1.2%—compared to improved DV-Hop, when t = 5; and reduces energy consumption by 27.88% compared to DV-Hop, reduces it by 16.9% compared to WCL, and slightly increases it—by 0.8%—when compared to improved DV-Hop, when t = 6, as depicted in Table 12 and Figure 11.

It has been found that when the threshold value rises, so does energy usage. The slight increase in energy consumption with our proposed algorithm is due to the extra step for enhancing localization accuracy. Thus, we suggest almost the same behavior for threshold values for minimizing energy consumption. Therefore, the proposed scheme is appropriate for WSNs because it has lower communication costs, improves localization accuracy, and increases the lifetime of WSNs by saving energy.

Table 13 provides a summary of the communication costs associated with the localization process for different numbers of nodes in the network. The energy consumption may also be affected by the number of hops (or relay steps) required to transmit a message from one node to another. A larger number of nodes may require more hops to transmit a message, leading to higher energy consumption. This can result in higher energy consumption for transmission, reception, and processing.

In general, it appears that energy consumption increases as the number of nodes increases for all four methods. However, the “Improved DV-Hop” and “Proposed” methods demonstrate consistently lower energy consumption than the other two methods, regardless of the number of nodes. This suggests that these methods may be more energy-efficient than the other methods.

The overall summary of this section is that the proposed algorithm has effectively reduced energy consumption and increased the lifetime of WSNs, although it does so with a slight increase in energy consumption due to the additional step taken to enhance location accuracy. This slight increase has been controlled by introducing a specific threshold in the last two phases. The number of hops required for message transmission can also affect energy consumption; fewer hops result in lower energy consumption. Overall, the proposed algorithm is a suitable choice for WSNs due to its reduced communication costs, improved localization accuracy, and increased energy efficiency.

#### 5.6.3. Computational Cost in Terms of Localization Time

The computational cost of the technique is defined by the time required to complete the localization procedure. The network’s size and scalability affect the amount of time it takes. In this simulation, 300 and 150 anchor nodes were randomly distributed in a 500 × 500 m^2^ space with a communication range of 100 m.

**Figure 12:**
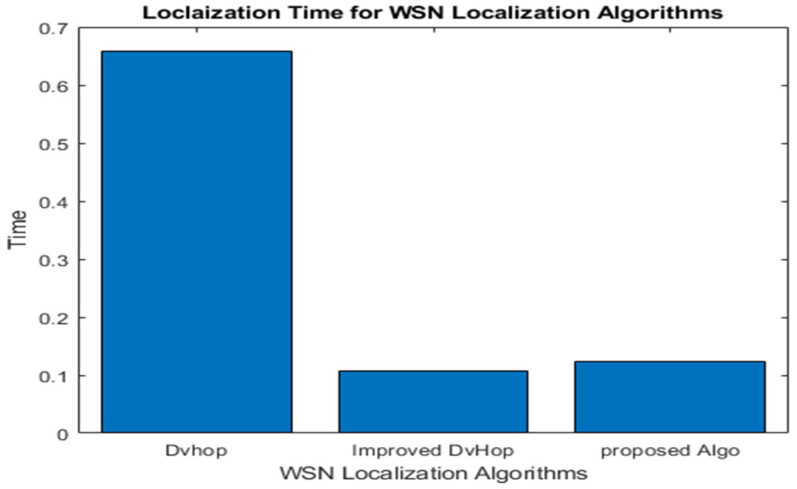
Localization time for localization algorithms.

It is observed that traditional DV-Hop consumes more time due to broadcasting packets to all networks. At the same time, the improved DV-Hop consumes little time due to limiting the broadcast to a specific threshold. Our proposed algorithm has better localization time than DV-Hop due to an extra correction step for the AHD; it consumes a little more time than improved DV-Hop, as shown in Table 14 and Figure 12. Thus, from the above performance analysis, it is confirmed that the proposed algorithm significantly improves the trade-off between localization accuracy and energy efficiency in WSNs. 

## 6. Conclusions and Future Work

The HCEDV-Hop algorithm was developed to enhance the traditional DV-Hop method, with the goal of improving localization accuracy while reducing energy consumption. Simulation results showed that the HCEDV-Hop algorithm consistently outperformed other algorithms, such as DV-Hop, WCL, improved DV-maxHop, and improved DV-Hop, in terms of localization accuracy and energy efficiency, with an average improvement of 81.36%, 77.99%, 39.72%, and 9.96%, respectively. In particular, the HCEDV-Hop algorithm demonstrated a significant decrease in energy consumption for message communication compared to traditional DV-Hop and WCL, at 27.88% and 17%, respectively. These findings demonstrate the efficacy of the proposed algorithm in improving the trade-off between localization accuracy and energy efficiency in wireless sensor networks. 

In future work, the HCEDV-Hop algorithm will be tested with mobile anchor nodes and evaluated in large-scale networks to improve localization accuracy by using an optimal path model for mobile anchor nodes. Mobile anchor nodes are essential in modern applications as they increase node lifespan and conserve energy in anisotropic WSNs.

## Figures and Tables

**Table 1:** Coordinates of anchor nodes.

Anchor ID	X Coordinate	Y Coordinate
1	29.63	19.335
2	24.074	29.108
3	8.938	19.338
4	19.144	22.799
5	13.711	24.57
6	45.926	47.122
7	16.546	46.217
8	9.797	40.045
9	27.994	47.507
10	19.926	15.123
11	33.680	48.236
12	46.440	19.567
13	19.979	3.788
14	12.635	3.335
15	2.0742	21.108

**Table 2:** Minimum hop of node “N” from every anchor.

Anchor ID	Average Hop Distance
1	1
2	1
3	4
4	3
5	3
6	2
7	4
8	4
9	2
10	3
11	1
12	2
13	4
14	5
15	5

**Table 3:** Average hop distance of all anchors.

Anchor ID	Average Hop Distance
1	29.261
2	34.467
3	23.946
4	24.786
5	22.386
6	18.692
7	21.370
8	19.704
9	22.533
10	22.199
11	21.887
12	25.697
13	21.960
14	21.832
15	29.229

**Table 4:** Refinement step in AHD for specific threshold “6”.

Anchor ID	Refinement Value of AHD
1	2.400
2	2.210
6	1.965
9	1.927
11	1.848
12	1.672

**Table 5:** Distance of node “*N*” from each anchor for specific threshold “6”.

Anchor ID	Distance (d_iu_)
1	35.23
2	35.23
6	35.23
9	35.23
11	47.901
12	47.901

**Table 6:** Parameter used in simulation.

Parameters for Simulation	Value
WSN area (m^2^)	500 * 500
Total nodes	300–500
Anchor nodes	50–175
Threshold (hops)	3–7
Radius (m)	100
Iterations	50

**Table 7:** Average localization error.

Localization Algorithms	Max.	Avg.	Min.	Std. Dev
DV-Hop (traditional)	1.8098	1.584926	1.407643	0.1358
WCL	1.3644	1.34262	1.32379	0.0144
Improved DV-maxHop	NA	0.49	NA	NA
Improved DV-Hop	0.3528	0.32808	0.3013	0.02489
HCEDV-Hop	0.30008	0.29540	0.28972	0.00361

**Table 8:** Comparison of localization error under various anchor node ratios.

No. of Anchors	DV-Hop	WCL	Improved DV-MaxHop	Improved DV-Hop	Proposed
50	1.808917	1.364458	0.7023	0.3528	0.294917
75	1.624204	1.346386	0.6119	0.3526	0.294601
100	1.611465	1.350904	0.4898	0.3464	0.2985
125	1.560510	1.332831	0.3971	0.3013	0.300085
150	1.496815	1.337349	0.3472	0.3088	0.2946
175	1.407643	1.323795	0.3265	0.3066	0.289721

**Table 9:** Comparison of localization error using various total numbers of nodes.

No. of Nodes	DV-Hop	WCL	Improved DV-Hop	Proposed
300	1.474095	1.162098	0.293621	0.288824
400	1.470506	1.184061	0.301444	0.291524
500	1.490854	1.192290	0.303121	0.289620
600	1.487330	1.195952	0.304770	0.289464
700	1.471756	1.204175	0.299806	0.286361
800	1.486177	1.198686	0.301160	0.294684

**Table 10:** Coverage area vs. average localization error.

Size of Coverage Area	DV-Hop	WCL	Improved DV-Hop	Proposed
100	1.276567	0.932440	0.29036	0.278921
300	1.59875	1.38567	0.338326	0.31768
500	1.67897	1.45876	0.472877	0.387654
700	1.8940	1.65792	0.499785	0.44654
900	2.2345	1.78659	0.51676	0.495432

**Table 11:** Communication cost of different algorithms.

Algorithm	Operation	Step 1	Step 2	TTRP
DV-Hop	Transmit packets	n×m	n	m×cavg(2n−1)+2n−m
	Get	$m\times \left(n-1\right)\times C_{avg}$	n−m	
	Total	$m\times n+\left(n-1\right)\times C_{avg}$	2n−m	
Improved DV-Hop	Transmit packets	n×m	n	m×cavg(2n−t)+2n−m−t
	Get	$m\times \left(n-t\right)\times C_{avg}$	n−m−t	
	Total	$m\times n+\left(n-t\right)\times C_{avg}$	2n−m−t	
Proposed Algorithm	Transmit packets	n×m	n	m×cavg(2n−t)+2n−m−t+REF
	Get	$m\times \left(n-t\right)\times C_{avg}$	n−m−t+REF	
	Total	$m\times n+\left(n-t\right)\times C_{avg}$	2n−m−t+REF	

**Table 12:** Comparison of the proposed algorithm, in terms of energy consumption, using various threshold values (anchor nodes = 150).

Threshold	DV-Hop	WCL	Improved DV-Hop	Proposed Algorithm
3	250,850	217,676	176,760	180,000
5	250,850	217,676	178,070	180,210
6	250,850	217,676	179,460	180,900

**Table 13:** Impact of node density on energy consumption.

No. of Nodes	DV-Hop	WCL	Improved DV-Hop	Proposed
100	31,400	24,200	16,270	16,396
200	143,077	91,542	86,357	86,573
300	250,850	217,676	178,070	180,210
400	371,066	356,070	338,990	339,202
500	581,456	561,987	540,840	541,032

**Table 14:** Comparison of localization times for various localization algorithms.

Localization Algorithms	Localization Time(s)
DV-Hop [7]	0.6572
Improved DV-Hop [35]	0.1078
HCEDV-Hop Algorithm	0.1237

## Data Availability

Data will be available on request.

## References

[1] Wang D., Zhang Q., Liu J. (2008). Partial network coding: Concept, performance, and application for continuous data collection in sensor networks. ACM Trans. Sens. Netw. (TOSN).

[2] Lu S.Y., Xie J.Y., Pang L.L. An improved WSN localization algorithm. Proceedings of the 2018 IEEE 2nd International Electrical and Energy Conference (CIEEC).

[3] Chowdhury T.J., Elkin C., Devabhaktuni V., Rawat D.B., Oluoch J. (2016). Advances on localization techniques for wireless sensor networks: A survey. Comput. Netw..

[4] Peng R., Sichitiu M.L. Angle of arrival localization for wireless sensor networks. Proceedings of the 2006 3rd Annual IEEE Communications Society on Sensor and Ad Hoc Communications and Networks.

[5] Sun Y., Zhang F., Wan Q. (2019). Wireless Sensor Network-Based Localization Method Using TDOA Measurements in MPR. IEEE Sens. J..

[6] Adewumi O.G., Djouani K., Kurien A.M. RSSI based indoor and outdoor distance estimation for localization in WSN. Proceedings of the 2013 IEEE International Conference on Industrial Technology (ICIT).

[7] Niculescu D., Nath B. (2003). DV based positioning in ad hoc networks. Telecommun. Syst..

[8] Mao G., Fidan B. (2019). Localization Algorithms and Strategies for Wireless Sensor Networks: Monitoring and Surveillance Techniques for Target Tracking: Monitoring and Surveillance Techniques for Target Tracking.

[9] Paul A.K., Sato T. (2017). Localization in Wireless Sensor Networks: A Survey on Algorithms, Measurement Techniques, Applications and Challenges. J. Sens. Actuator Netw..

[10] Nemer I., Sheltami T., Shakshuki E., Abu Elkhail A., Adam M. (2020). Performance evaluation of range-free localization algorithms for wireless sensor networks. Pers. Ubiquitous Comput..

[11] Gayan S., Dias D. Improved DV-Hop algorithm through anchor position re-estimation. Proceedings of the 2014 IEEE Asia Pacific Conference on Wireless and Mobile.

[12] Fang W., Yang G. Improvement based on DV-Hop localization algorithm of wireless sensor network. Proceedings of the 2011 international conference on mechatronic science, electric engineering and computer (MEC).

[13] Liu J., Wang W., Shang Y. An improving localization algorithm for wireless sensor networks based on DV-Hop. Proceedings of the 2012 International Conference on Measurement, Information and Control.

[14] Yu W., Li H. An improved DV-Hop localization method in wireless sensor networks. Proceedings of the 2012 IEEE international conference on computer science and automation engineering (CSAE).

[15] Chen Y., Li X., Ding Y., Xu J., Liu Z. An improved DV-Hop localization algorithm for wireless sensor networks. Proceedings of the 2018 13th IEEE conference on industrial electronics and applications (ICIEA).

[16] Zhang B., Ji M., Shan L. A weighted centroid localization algorithm based on DV-hop for wireless sensor network. Proceedings of the 2012 8th international conference on wireless communications, networking and mobile computing.

[17] Song G., Tam D., Xue Y., Liu B. RDV-Hop localization algorithm for randomly deployed wireless sensor networks. Proceedings of the International Conference on Software Intelligence Technologies and Applications & International Conference on Frontiers of Internet of Things 2014.

[18] Fang X. (2015). Improved DV-Hop Positioning Algorithm Based on Compensation Coefficient. J. Softw. Eng..

[19] Tomic S., Mezei I. (2015). Improvements of DV-Hop localization algorithm for wireless sensor networks. Telecommun. Syst..

[20] Xiang M., Wang S., Yang Y. (2016). Improved DV-Hop localization algorithm based on threshold mechanism and distance correction for WSN. J. Transducer Technol..

[21] Mass-Sanchez J., Ruiz-Ibarra E., González J.C., Espinoza-Ruiz A., Castro L.A. (2016). Weighted Hyperbolic DV-Hop Positioning Node Localization Algorithm in WSNs. Wirel. Pers. Commun..

[22] Wang J., Gao Y., Yin X., Li F., Kim H.-J. (2018). An Enhanced PEGASIS Algorithm with Mobile Sink Support for Wireless Sensor Networks. Wirel. Commun. Mob. Comput..

[23] Qiao X., Chang F., Ling J. (2019). Improvement of Localization Algorithm for Wireless Sensor Networks Based on DV-Hop. Int. J. Online Biomed. Eng. (iJOE).

[24] Khan M.A., Khan M.A., Rahman A.U., Malik A.W., Khan S.A. (2019). Exploiting cooperative sensing for accurate target tracking in industrial Internet of things. Int. J. Distrib. Sens. Netw..

[25] Xue D. (2019). Research of localization algorithm for wireless sensor network based on DV-Hop. EURASIP J. Wirel. Commun. Netw..

[26] Wang J., Hou A., Tu Y., Yu H. (2020). An Improved DV-Hop Localization Algorithm Based on Selected Anchors. Comput. Mater. Contin..

[27] Bhat S.J., Santhosh K. Priority based localization for anisotropic wireless sensor networks. Proceedings of the 2020 IEEE International Conference on Distributed Computing, VLSI, Electrical Circuits and Robotics (DISCOVER).

[28] Bhat S.J., Santhosh K. (2020). Is localization of wireless sensor networks in irregular fields a challenge?. Wirel. Pers. Commun..

[29] Li X., Wang K., Liu B., Xiao J., Han S. (2020). An improved range-free location algorithm for industrial wireless sensor networks. EURASIP J. Wirel. Commun. Netw..

[30] Shahzad F., Sheltami T., Shakshuki E. (2016). DV-maxHop: A fast and accurate range-free localization algorithm for anisotropic wireless networks. IEEE Trans. Mob. Comput..

[31] Gupta A., Mahaur B. (2021). An improved DV-maxHop localization algorithm for wireless sensor networks. Wirel. Pers. Commun..

[32] Messous S., Liouane H., Cheikhrouhou O., Hamam H. (2021). Improved Recursive DV-Hop Localization Algorithm with RSSI Measurement for Wireless Sensor Networks. Sensors.

[33] Kumar S., Lobiyal D.K. (2016). Novel DV-Hop localization algorithm for wireless sensor networks. Telecommun. Syst..

[34] Goyat R., Rai M.K., Kumar G., Saha R., Kim T.-H. (2019). Energy Efficient Range-Free Localization Algorithm for Wireless Sensor Networks. Sensors.

[35] Kaur A., Kumar P., Gupta G. (2020). Improving DV-Hop-Based Localization Algorithms in Wireless Sensor Networks by Considering Only Closest Anchors. Int. J. Inf. Secur. Priv..

[36] Liu X. (2020). Research on WSN Node Localization Algorithm Based on RSSI Iterative Centroid Estimation. Teh. Vjesn. Tech. Gaz..

[37] Zhou G., He T., Krishnamurthy S., Stankovic J.A. (2006). Models and solutions for radio irregularity in wireless sensor networks. ACM Trans. Sens. Netw..

[38] The Math Works, Inc. (2020). MATLAB, Version 2020a.

[39] He T., Huang C., Blum B.M., Stankovic J.A., Abdelzaher T. Range-free localization schemes for large scale sensor networks. Proceedings of the 9th annual international conference on Mobile computing and networking.

[40] Peng B., Li L. (2015). An improved localization algorithm based on genetic algorithm in wireless sensor networks. Cogn. Neurodynamics.

[41] Wang C., Chen J., Sun Y., Shen X. (2009). Wireless sensor networks localization with isomap. 2009 IEEE International Conference on Communications.

[42] Kaur A., Kumar P., Gupta G. (2017). Analysis on DV-Hop Algorithm and its variants by considering threshold. J. Telecommun. Electron. Comput. Eng. (JTEC).

[43] Khelifi F., Bradai A., Benslimane A., Kaddachi M.L., Atri M. Energy-saving performance of an improved DV-hop localization algorithm for wireless sensor networks. Proceedings of the GLOBECOM 2017–2017 IEEE Global Communications Conference.

[44] Kotwal S.B., Verma S., Abrol R.K. (2012). RSSI WSN localization with Analysis of Energy Consumption and Communication. Int. J. Comput. Appl..

[45] Baba A.I., Wu F. (2015). Energy-Accuracy Trade-off in Wireless Sensor Network Localization. Int. J. Handheld Comput. Res..

